# Forensic toxicokinetics of difenidol hydrochloride in vivo verified by practical lethal cases

**DOI:** 10.1038/s41598-023-38246-y

**Published:** 2023-07-11

**Authors:** Weifen Niu, Tongxi Ren, Yandan Wang, Zheyu Wang, Chao Zhang, Juan Jia, Zhiwen Wei, Hongliang Su, Zhongyuan Guo, Xiangjie Guo, Keming Yun

**Affiliations:** 1 School of Forensic Medicine, Shanxi Medical University, Jinzhong, 030600 Shanxi People’s Republic of China; 2 Shanxi Key Laboratory of Forensic Medicine, Jinzhong, 030600 Shanxi People’s Republic of China; 3 Key Laboratory of Forensic Toxicology of Ministry of Public Security, Jinzhong, 030600 Shanxi People’s Republic of China; 4 Institute of Forensic Science of Tianjin Public Security Bureau, Tianjin, 300384 People’s Republic of China

**Keywords:** Medicinal chemistry, Toxicology, Pharmacokinetics

## Abstract

A gas chromatography-mass spectrometry (GC–MS) method for the determination of difenidol hydrochloride in biological specimens has been developed. The method exhibited excellent recovery (> 90%) and precision (RSD < 10%), and the LOD was 0.05 μg/mL or μg/g, which met the requirements of bioanalytical method. Through the animal model of the forensic toxicokinetics, the dynamic distribution, postmortem redistribution (PMR) and stability in specimen preservation process of difenidol in animals were studied. The experimental results showed that after intragastric administration, the difenidol’s concentrations in the heart-blood and various organs increased over time except stomach, and then decreased gradually after reaching the peaks of concentration. The toxicological kinetics equation and toxicokinetic parameters were established by processing the data of the mean drug concentration of difenidol changing with time. In PMR experiment, the concentrations of difenidol in some organs closer to the gastrointestinal tract (heart-blood, heart, liver, lung, kidney, and spleen) changed significantly at different time points. But the concentration of difenidol in brain tissues which were far away from the gastrointestinal tract and muscles with larger overall mass was relatively stable. PMR of difenidol was therefore confirmed. Thus, the effect of PMR on the concentration of difenidol in the specimens should be considered in cases involving difenidol poisoning or death. Furthermore, the stability of difenidol in heart-blood samples from poisoned rats was investigated at various time points and under different preservation conditions (20 °C, 4 °C, − 20 °C and 20 °C (1% NaF)) for a period of two months. Difenidol was stable and did not decompose in the preserved blood. Therefore, this study provided the experimental basis for the forensic identification of the cases of difenidol hydrochloride poisoning (death). PMR has been verified by practical lethal cases.

## Introduction

As an antiemetic agent widely used in Latin America and Asia, difenidol hydrochloride (1,1-diphenyl-4-piperidino-1-butanol hydrochloride) was first approved in the United States in 1967 to treat vomiting and vertigo. Difenidol hydrochloride has good anti-vertigo effect and less adverse reactions^1–4^, so it has been widely used in clinical practice. Difenidol is used to inhibit dizziness and antinausea due to its expansion of spasticity, increase blood flow of vertebral artery and basilar artery, regulate vestibular function, inhibit abnormal impulse of vestibular nerve and pharmacological function of vomiting centres^5,6^. However, in the last 20 years, there have been a number cases of suicides and accidental poisonings related to difenidol in China^7–19^. The main symptoms of difenidol poisoning include dry mouth, irritability, hallucinations, headache, euphoria, and temporary hypotension. At high doses, there is H1 receptor antagonists poisoning, which the clinical manifestations are central nervous system inhibition, then excitation and then re-inhibition.^9,10,16^. Toxic patients often die of respiratory failure due to inhibition of the respiratory center, hypotensive shock, or arrhythmia.

The most previous studies focused on difenidol’s pharmacology, pharmacokinetics, and clinical application, but they failed to study on the forensic toxicokinetics of difenidol. In fatal poisonings, explanations for possible causes of death often rely on the drug concentrations in vivo. However, there is no widely accepted doses of difenidol in human blood of poisonous or lethal cases. But in fact, postmortem drug concentrations may vary depending on the sampling site and the interval between death and postmortem specimen collection. These changes, which depend on site and time, are known as postmortem redistribution (PMR) in forensic science^20,21^. In view of the effect of PMR, cause-of-death interpretation may be complicated by comparing postmortem concentrations with drug concentrations at the time of death, making it difficult to determine whether death was attributable to an overdose of difenidol. Therefore, it is necessary to study in detail the dynamic distribution and PMR of difenidol hydrochloride.

In this paper, the animal model of forensic toxicokinetics for difenidol was developed. The dynamic distribution, PMR and stability in specimen preservation process of difenidol in rats were studied using developed GC–MS method. This research provides the experimental basis for the forensic identification of the cases of difenidol hydrochloride poisoning (death). PMR has been verified by practical lethal cases.

## Materials and methods

### Chemicals

Difenidol hydrochloride standard was provided by Institute of Forensic Science, Ministry of Public Security, China. Difenidol hydrochloride tablets were purchased from Dalian Tianyu Haibin Pharmaceutical Co., LTD. (lot Number: 050703). Proadifen hydrochloride (SKF_525A_) used as the internal standard was obtained from Standard Technology Development Co. Ltd, China. All standard substances were prepared into a solution of 1.0 mg/mL with methanol as solvent, stored at 4 °C, and diluted as needed. Other chemicals were analytically pure and purchased from Sigma.

### Instrumental parameters

The Thermo Fisher Scientific TRACE DSQ system (USA) was used for GC–MS analysis. The extract (1 μL) was injected into a DB-5 capillary column (30 m × 0.25 mm × 0.25 μm, J&W, Folsom, CA). Column temperature program was as follows: the initial temperature was 180 °C, held for 1 min, then rose to 280 °C at a rate of 10 °C/min, and lastly held at 280 °C for 2 min. Injection port temperature was 250 °C, and the split injection mode was adopted. Carrier gas was helium with a constant flow of 1.5 mL/min. EI source settings were: voltage: 70 eV, source temperature: 200 °C, transfer line temperature: 250 °C, and Quality range: 50–650. The MS was operated in the selected ion monitoring mode.

### Animal experiments

The experimental animals were male Wistar rats weighing 200 ± 10 g, which were provided by the Laboratory Animal Center of Shanxi Medical University, China. Before the experiment, the rats were fasted for 12 h. The experiment was approved by Institutional Animal Care and Use Committee of Shanxi Medical University and carried out in accordance with current relevant laws and regulations in China. Authors complied with the ARRIVE guidelines.

#### Dynamic distribution of difenidol in poisoned rats

The dynamic distribution experiment of difenidol was carried out in 24 rats with lethal dose 50% (LD_50_) (500 mg/kg) difenidol hydrochloride by means of intragastric administration. 24 rats were divided into 8 groups (n = 3) and were sacrificed by cervical dislocation after 1 h, 1.5 h, 1.86 h, 5.02 h, 11.01 h, 20.16 h, 29.31 h, 47.61 h of administration, respectively. Specimens of heart, liver, spleen, lung, kidney, brain, stomach, and heart-blood were collected immediately after the rats’ death.

#### PMR of difenidol in poisoned rats

In the experiment of PMR, 21 rats were given 1.6 × LD_50_ (800 mg/kg) difenidol hydrochloride by means of intragastric administration. All rats were sacrificed by cervical dislocation 4 h after administration. The autopsy was performed at 0 h, 4 h, 8 h, 16 h, 24 h, 48 h, 72 h (n = 3 for each group) after animals’ death, and the specimens of heart, liver, spleen, kidney, lung, brain, muscle (left leg) and heart-blood were collected respectively. All specimens were then treated, and GC–MS detected the concentration of difenidol in each specimen.

#### Stability of difenidol in specimen preservation process

Four rats were fasted overnight before the experiments, and they were given 148 mg/kg difenidol hydrochloride by means of intragastric administration at a constant speed within 5 min. Rats were sacrificed by cervical dislocation after 2 h, and the heart-blood specimens were collected with heparin as anticoagulation. The heart-blood was divided into four equal parts and stored at temperatures of 20 °C, 4 °C, − 20 °C, and 20 °C (1% NaF), respectively. The concentration of difenidol was detected on 0 h, 1 day, 4 days, 10 days, 30 days, and 60 days.

### Extraction procedure

The extraction procedure was performed according to the references^22^ published by this research group. First, 2 mL deionized water was added to each solid specimens (1 g), then the samples were homogenized with a high-speed tissue homogenizer and 20 μL (1.0 mg/mL) internal standard SKF_525A_ solution was added to the homogenate. 20 μL (1.0 mg/mL) SKF_525A_ solution was added to 2 mL of heart-blood. Next, 1.0 mL HCl (10%) was added to the above samples for acid hydrolysis, and pH value of the sample solutions were lastly adjusted by adding 1.0 mL NaOH (2.0 M). The samples were then extracted with diethyl ether (5.0 mL), oscillated for 15 min and centrifuged for 15 min (1800 rpm). The diethyl ether extract was concentrated under nitrogen flow at 40 °C, and the residue was dissolved with 100 μL ethanol for GC–MS analysis according to the instrumental parameters mentioned above. Some specimens, such as the stomach, had a high concentration of difenidol and must be appropriately diluted before analysis.

### Method validation

For blood and liver, validation was partly performed according to the references^23,24^ for sensitivity, limit of detection (LOD), low limit of quantification (LOQ), linearity, precision, and recovery. Spiked samples were prepared by adding difenidol hydrochloride to blank blood or blank liver to a final concentration of 1.0, 2.0, 4.0, 8.0, 16.0, 20.0, 60.0, 100.0, 120.0, 160.0, 200.0, 400.0 μg/mL or μg/g, respectively, and 10.0 μg/mL (ug/g) internal standard. The sensitivity of the method for each matrix was assessed by determining the LOD and LOQ from the corresponding calibration plot as the signal to noise ratios of 3:1 and 10:1^25^, respectively. The retention times of difenidol required < 2% of error in various specimens and the spiked samples of counterpart; and moreover, the relative abundance ratios of ions in various specimens to the spiked have to be < 20% by GC–MS.

Precision and recovery were evaluated at three quality control (QC) concentrations of difenidol hydrochloride in five replicates respectively. QC samples were prepared before each assay by spiking blank samples with the difenidol standard solution at the determined concentrations. Intra-assay and inter-assay (3 days) precision were represented by relative standard deviations (RSD, %), which were supposed to be < 15%. The recovery rates of each matrix after extracting processes were calculated via determining concentration of difenidol hydrochloride in the spiked samples.

### Statistical analysis

Statistical analyses were performed with the SPSS program 11.5 and the data was represented by “mean ± standard deviation (SD)”. As with all statistical measures, a *P* value < 0.05 was considered significant in this study.

### Approval for animal experiments

Animal experiments were conducted according to the guidelines of the Declaration of Helsinki, and approved by the Institutional Animal Care and Use Committee of Shanxi Medical University, China. The study was approved by the ethics committee of Shanxi Medical University, Shanxi, China (permit number: 2020GLL022). The authors complied with the ARRIVE guidelines.

### Approval for human experiments

Human specimens’ experiments were approved by the ethics committee of Shanxi Medical University, Shanxi, China (permit number: 2020GLL022) and conducted according to the principles expressed in the Declaration of Helsinki. The informed consent was obtained from their legal guardians of the deceased.

## Results and discussion

### GC–MS method of difenidol hydrochloride

A sensitive GC–MS method was successfully developed and validated for the analysis of difenidol hydrochloride. GC–MS total ion current chromatogram and mass spectrogram are shown in the Fig. S1–S5. The retention time (*R*_t_) of difenidol hydrochloride and internal standard were 10.25 and 9.25, respectively. The MS was operated in the selected ion monitoring mode: the characteristic ion mass-to-charge ratios (m/z) of difenidol hydrochloride were 77, 98 and 232, and the internal standard were 86, 99 and 353. The ratio of peak area for difenidol hydrochloride to internal standard was found to be linearly proportional to difenidol hydrochloride concentration in the range of 1.0–400.0 μg/mL or μg/g, based on the linear regression equation of *Y* = 0.11*c* − 2.12 for blood and *Y* = 0.047*c* + 0.31 for liver, with a linear coefficient of 0.997 and 0.996, respectively. The LOD was 0.05 μg/mL or μg/g. The recovery was more than 90% and precision (RSD) was less than 10% for all the analytes. The method validation results are shown in Table 1. In a word, all validation results of GC–MS have met the requirements of bioanalytical method.

**Table 1:** Recovery and precision of difenidol (n = 5).

Biological sample	Quality controls (QC) (μg/mL or μg/g)	Detected (μg/mL or μg/g)	Recovery (%)	Precision (RSD, %)	
				Intra-assay	Inter-assay
Blank blood	1.0	0.97 ± 0.03	97.00 ± 1.56	5.95	6.23
	8.0	7.82 ± 0.37	97.75 ± 3.91	4.32	5.46
	16.0	15.20 ± 0.73	95.38 ± 2.94	3.83	4.22
Blank liver	2.0	1.97 ± 0.04	98.50 ± 1.74	5.83	3.53
	20.0	19.01 ± 0.32	95.05 ± 4.64	4.63	4.77
	40.0	38.62 ± 0.66	96.55 ± 3.42	4.02	5.22

### Dynamic distribution of difenidol in poisoned rats

**Figure 1:**
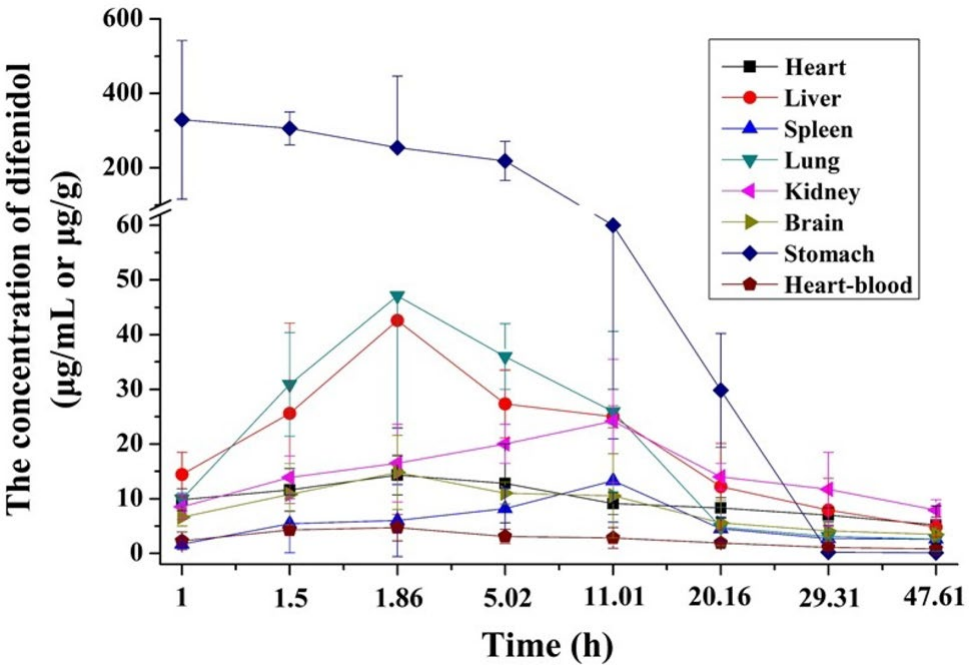
The concentrations of difenidol in different specimens at different time points after intragastric administration. Data were represented by mean ± SD.

After intragastric administration, difenidol hydrochloride was unevenly distributed in heart-blood and organs, and its concentrations varied over time. The concentrations of difenidol in various specimens at different time points were presented in Fig. 1. Difenidol hydrochloride was rapidly absorbed and then distributed into well-perfused tissues via the circulatory system. The difenidol’s concentrations in the heart-blood and different organs increased over time except stomach, and decreased gradually after reaching the peaks of concentration. The above experimental results showed that the distribution of difenidol hydrochloride in the rats was dynamic.

The data of the mean drug concentration of difenidol changing with time were processed by WinNonlin pharmacokinetics software. The results showed that the toxicological kinetics of difenidol was characterized by first-order kinetics, in line with the two-compartment open model. The toxicological kinetics equation was *C*_t_ = 7.59e^−0.79*t*^ + 3.26e^−0.04*t*^ + 10.85e^−1.70*t*^, and toxicokinetic parameters of difenidol in organs and heart-blood were shown in Table 2. The models estimated that the concentration in the heart-blood reached its peak at 1.95 h (T_max_) after administration and the peak blood concentrations (C_max_) was 4.68 μg/mL.

**Table 2:** Toxicokinetic parameters of difenidol.

Biological sample	T_max_ (h)	C_max_ (μg/mL or μg/g)	t_1/2k01_ (h)	t_1/2k10_ (h)	t_1/2α_ (h)	t_1/2β_ (h)
Heart	2.85	12.93	0.48	27.84	–	–
Liver	3.43	33.64	0.84	11.79	–	–
Spleen	8.14	11.49	5.48	5.48	–	–
Lung	2.63	47.08	0.40	6.86	–	–
Kidney	6.55	22.95	1.63	21.51	–	–
Brain	3.41	12.86	0.73	16.08	–	–
Stomach	2.21	326.90	1.53	1.53	–	–
Heart-blood	1.95	4.68	0.41	9.58	0.89	20.67

T_max_, Time of peak; C_max_, concentration of peak; t_1/2k01_, absorption half-life; t_1/2k10_, elimination half-life; t_1/2α_, half-life of distributed phase; t_1/2β_, half-life of elimination phase.

Difenidol had excellent liposolubility^26^ and moderate plasma protein binding (42–70%)^27^. The apparent distribution volume (Vd) was 68.72 L/kg, which was far exceeding the total body fluid of the rats. The results indicated that the blood concentration was low and easy to be absorbed by tissues. Difenidol hydrochloride was metabolized slowly with an elimination half-life of 9.58 h. It was mainly metabolized in liver and eliminated via kidneys. After 47.61 h, it was almost eliminated in the most organs and heart-blood, but the concentration in the kidney was still significantly higher than that in other organs.

### PMR of difenidol in poisoned rats

As the time interval between death and specimen collection increased, postmortem drug concentrations may vary. Many drugs were redistributed after death (i.e., PMR)^28^. The research results showed that the poisons with alkaline, lipophilic, and large Vd were more likely to occur PMR^21,29–31^. Difenidol with excellent liposolubility^26^, fast oral absorption, slow distribution, and elimination, should be more prone to occur PMR.

**Figure 2:**
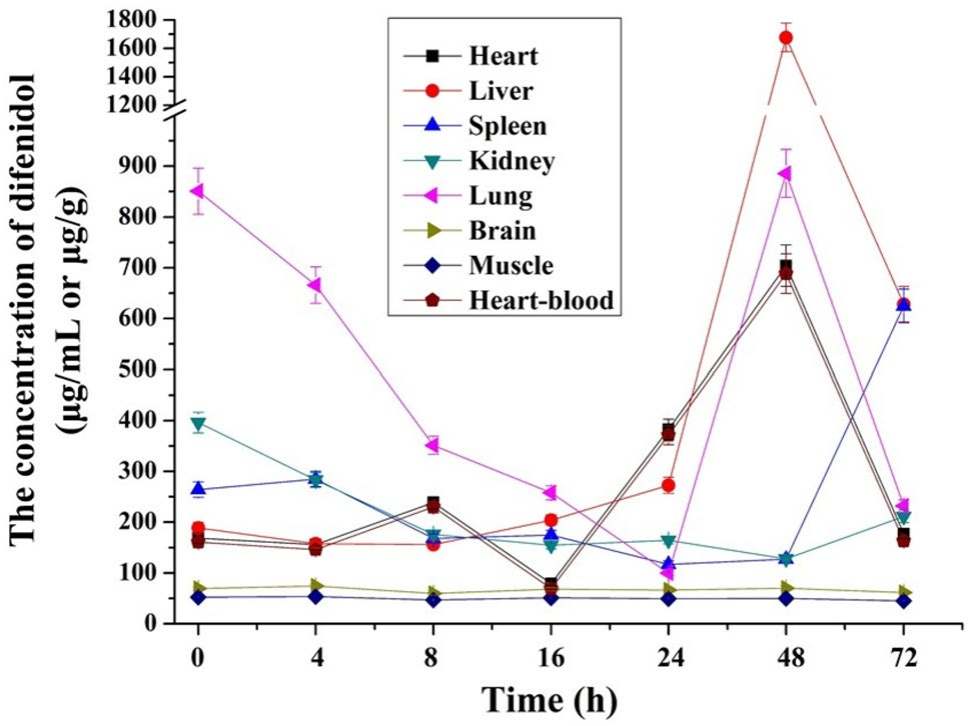
PMR of difenidol in poisoned rats. Data were represented by mean ± SD.

The autopsy was carried out at seven different time points and various specimens were collected, and the concentrations of difenidol in various specimens were detected separately. The results were displayed in Fig. 2, in brain tissues and muscles, there was no statistically significant difference (*p* > 0.05) in contents at different time points. As to other specimens, the differences of difenidol’s concentrations at some time points were significant (*p* < 0.05 compared with the concentrations of difenidol at 0 h). As showed in Fig. 2, the concentration varied greatly in heart-blood, heart, liver and lung: which were stable at first in heart-blood, heart and liver of rats, then increased significantly and then decreased; The concentration in kidney showed a trend of decreasing first and then increasing; The lungs showed the highest concentration level of difenidol at 0 h time point and then decreased gradually, and then increased sharply to a higher concentration at the 48 h time point and then decreased again. The spleen first descends, then stabilizes and finally rises. Moreover, the concentration of difenidol in the liver was highest compared to other tissues and blood after 40 h. Difenidol undergo PMR in the rats poisoned by means of intragastric administration. The concentrations of difenidol in brain tissues and muscles varied conservatively within 72 h, and therefore combined with the known postmortem time, the ratios of difenidol concentrations in other specimens to the concentrations in brain tissues or muscles can be used to infer the concentrations in each specimen at the time of death.

In recent years, several hypotheses had been proposed to explain the mechanisms underlying PMR of drugs: the distribution through the choroid plexus, diffusion toward the surrounding organs transparietally, cell autolysis and putrefaction^30–34^, etc. In this study, difenidol hydrochloride was administered intragastrically, and stomachs tend to serve as “drug reservoirs”. The above experimental results showed that the drug concentration in organs far away from the gastrointestinal tract and tissues with larger overall mass was relatively stable, and the PMR not obvious. At room temperature, the concentration of difenidol in the viscera changed significantly at about 48 h, which could be because the tissues had begun to decay at this time, and the change could be related to the role of microorganisms, making the binding drugs become free type or the integrity of the biofilm destroyed, thus leading to the increase of the drug concentration in the tissues and blood^35,36^. Therefore, PMR should be considered in the determination of difenidol poisoning deaths and causes of death. In the forensic medical identification of death cases caused by difenidol, in addition to the routine specimens such as heart-blood, liver and stomach contents, the tissues and organs with relatively less obvious changes in the concentration of difenidol, such as brain and muscle should be examined.

### Stability of difenidol in specimen preservation process

**Figure 3:**
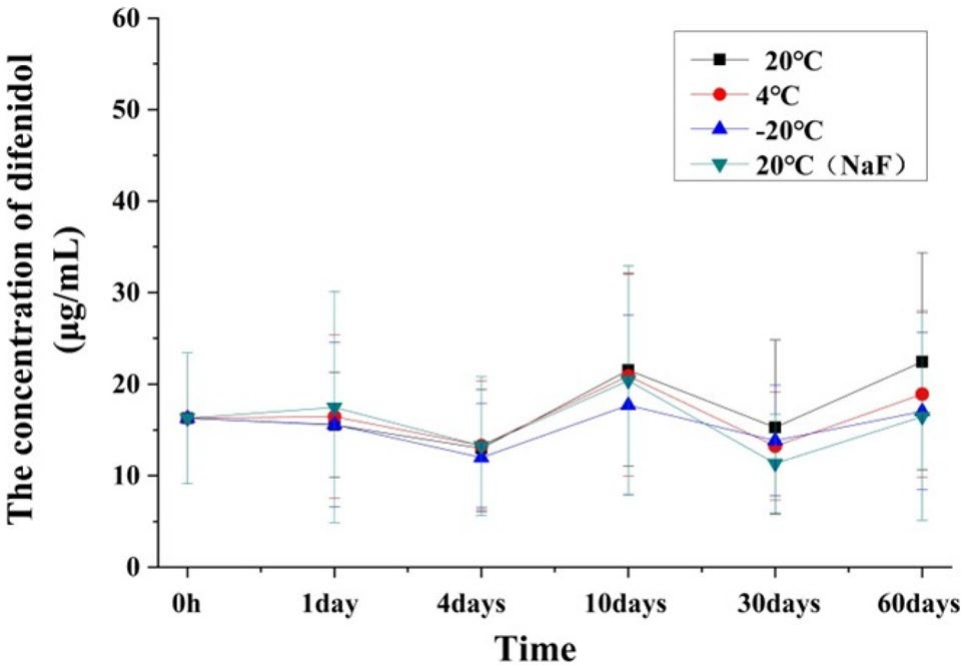
The concentrations of difenidol in heart-blood at different time points under different preservation condition (20 °C, 4 °C, − 20 °C and 20 °C (1% NaF)) within 2 months.

Stability was a dynamic process in which the concentration of difenidol changes with time during specimen preservation process^37^. The experimental results were shown in the Fig. 3. Compared with 0 h, there was no statistically significant changes for the concentration of difenidol in heart-blood of poisoned rats at various time point under different preservation condition (20 °C, 4 °C, − 20 °C and 20 °C (1% NaF)) within 2 months (*p* > 0.05). The experimental results indicated that difenidol was stable and did not decompose in the preserved blood. Therefore, in the forensic medical evaluation of death from difenidol poisoning, the decomposition in the stored blood should not be considered within 2 months.

### Application in practical lethal cases

The developed GC–MS method was successfully applied to the detection and quantification of difenidol in four practical suspected cases of difenidol hydrochloride poisoning, and the results were shown in Table 3. In the Case 1, 2, and 3, the concentration of difenidol in heart-blood was between 17 and 43 μg/mL. The blood sample in the Case 4 of decomposed cadaver was failed to be collected, so the defenidol in liver was used for quantification and the concentration was 1.78 mg/g. In routine practice, the reference values are used to assess the contribution of difenidol by the forensic pathologists and toxicologists in the fatalities, and the values might differ in different cases of difenidol intoxication. It was reported that the concentration of difenidol in blood ranged from 2.71 to 83.1 μg/mL in different cases of death and in liver ranged from 25.69 to 235 μg/g^10–12^. The determination results of difenidol in heart-blood in the above three cases were all in this range. However, the concentration of difenidol in the liver (1.78 mg/g) was beyond the range reported. According to the experimental results of this study on PMR of poisoned rats that with higher concentrations in the liver after 40 h than in other tissues (Fig. 2), difenidol should undergo PMR.

**Table 3:** Cases reports.

	Brief case summary	Specimens	Determination results
1	The deceased had a meal with his friends. During the meal, he felt unwell and was rushed to the hospital. After emergency treatment, he died. After investigation, he died of poisoning	Heart-blood	17.31 μg/mL
2	Unknown	Heart-blood	42.88 μg/mL
3	Unknown	Heart-blood	35.90 μg/mL
4	The deceased was found in a white car in a riverside parking lot. The cadaver was decomposed and blood sample was not available	Liver	1.78 mg/g

The large postmortem drug concentration differences may be related to many factors^38^: sampling time, elapsed time after death, underlying diseases, individual variations, etc. Moreover, the combination of other drugs and difenidol may deepen the inhibitory effect on the respiratory and circulation centers. A synergistic effect of difenidol and other drugs may also exist^38,39^. Therefore, in cases involving difenidol death, in order to accurately determine the cause of death, more relevant specimens should be collected for testing, and multiple factors should be considered comprehensively, including but not limited to postmortem redistribution, disease, drug combination, etc.

## Conclusion

In summary, a sensitive, accurate and rapid GC–MS method was developed for qualitative and quantitative analyses of difenidol in blood and liver tissue, and the LOD is 0.05 μg/mL or μg/g. By studying the forensic toxicokinetics, difenidol’s concentrations varied according to the organ and time, and difenidol undergo PMR in the rats poisoned by means of intragastric administration. So, PMR should be considered in the determination of difenidol poisoning deaths and causes of death. And the study has been verified by practical lethal cases. This study is supposed to facilitate the diagnosis of difenidol poisoning.

## Supplementary Information

Supplementary Figures.

## Data Availability

All data generated or analyzed during this study are included in this published article [and its supplementary information files].
