# Supplementary material for: Forensic toxicokinetics of difenidol hydrochloride in vivo verified by practical lethal cases
Source: Sci Rep. 2023 Jul 11;13:11190. doi: 10.1038/s41598-023-38246-y (PMC10336003; doi:10.1038/s41598-023-38246-y)
Supplement: Supplementary file 1 — Supplementary Figures. [file 41598_2023_38246_MOESM1_ESM.docx]

**Supplementary Information**

**Forensic Toxicokinetics of Difenidol Hydrochloride *in vivo* Verified by Practical Lethal Cases**

Weifen Niu^1,2,3, a^, Tongxi Ren^4, a^, Yandan Wang^1,2,3^, Zheyu Wang^1,2,3^, Chao Zhang^1,2,3^, Juan Jia^1,2,3^, Zhiwen Wei^1,2,3^, Hongliang Su^1,2,3^, Zhongyuan Guo^1,2,3^, Xiangjie Guo^1,2,3,^*, Keming Yun^1,2,3,^*

^1^ School of Forensic Medicine, Shanxi Medical University, Jinzhong 030600, Shanxi, P. R. China

^2^ Shanxi Key Laboratory of Forensic Medicine, Jinzhong, 030600, Shanxi, China

^3^ Key Laboratory of Forensic Toxicology of Ministry of Public Security, Jinzhong, 030600, Shanxi, China

^4^ Institute of Forensic Science of Tianjin Public Security Bureau, Tianjin, 300384, P.R. China

^a^ These authors contributed equally to this work

*Author to whom correspondence should be addressed. Email: yunkeming5142@163.com

**Contents**

**Supplementary Figures**

**Fig. S1** GC-MS total ion current chromatograms of blank blood added internal standard of proadifen hydrochloride (SKF_525A_) (10.0 μg/mL)

**Fig. S2** GC-MS total ion current chromatogram of spiked blood with difenidol (10.0 μg/mL) and SKF_525A_ (10.0 μg/mL).

**Fig. S3** GC-MS total ion current chromatogram of blood in difenidol poisoned rats added SKF_525A_ (10.0 μg/mL).

**Fig. S4** Mass spectrogram and chemical structure of difenidol

**Fig. S5** Mass spectrogram and chemical structure of SKF_525A_


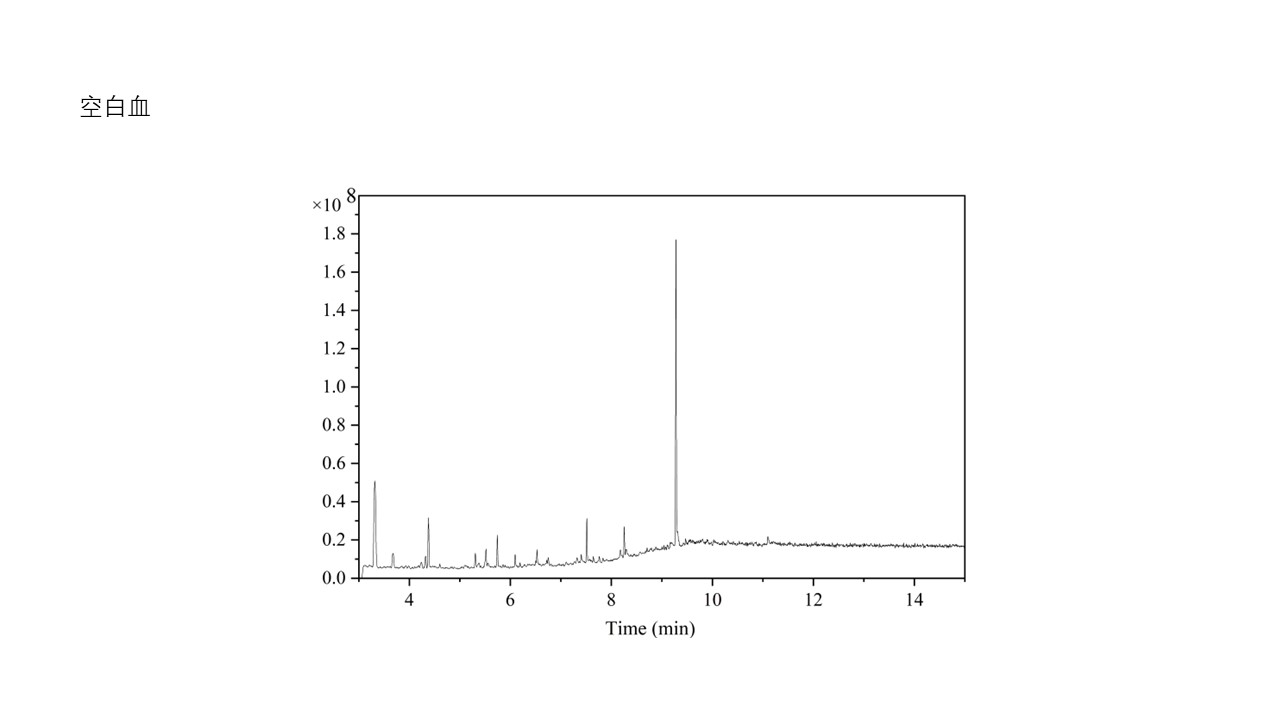


**Fig. S1** GC-MS total ion current chromatograms of blank blood added internal standard of proadifen hydrochloride (SKF_525A_) (10.0 μg/mL). SKF_525A_ *R*_t_: 9.28min


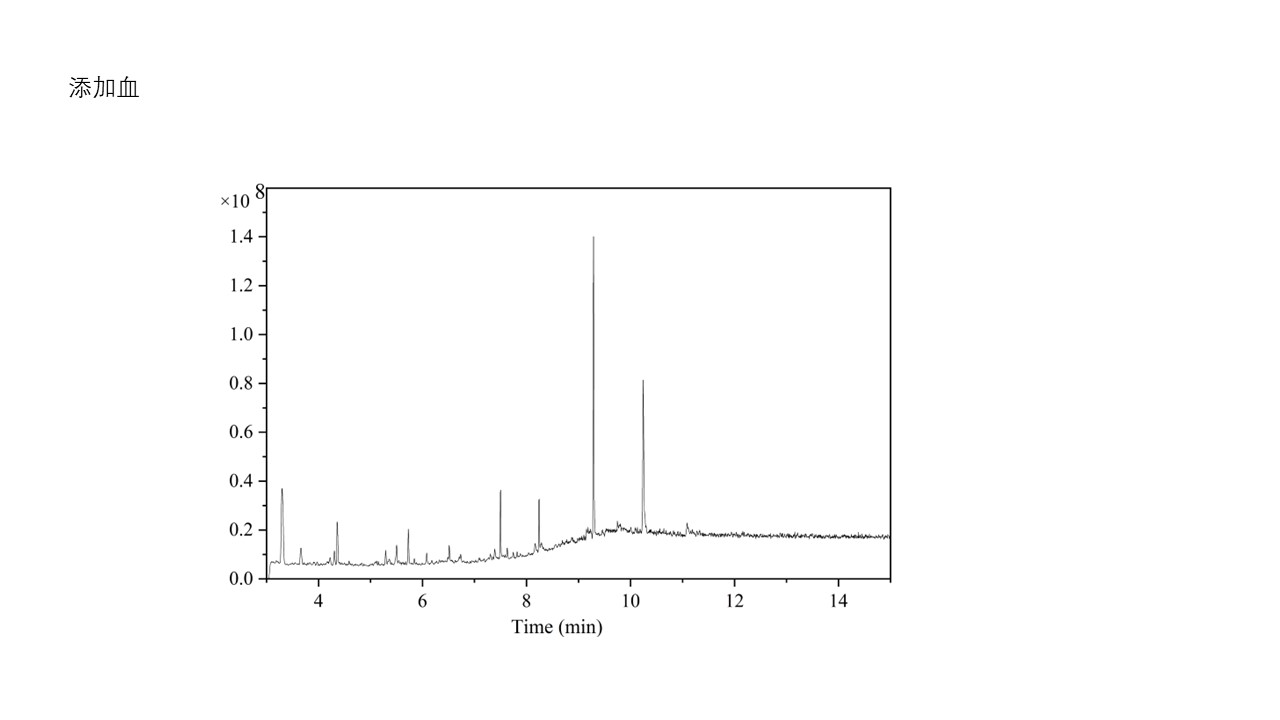


**Fig. S2** GC-MS total ion current chromatogram of spiked blood with difenidol (10.0 μg/mL) and SKF_525A_ (10.0 μg/mL). Difenidol *R*_t_: 10.25min; internal standard SKF_525A_ *R*_t_: 9.25min


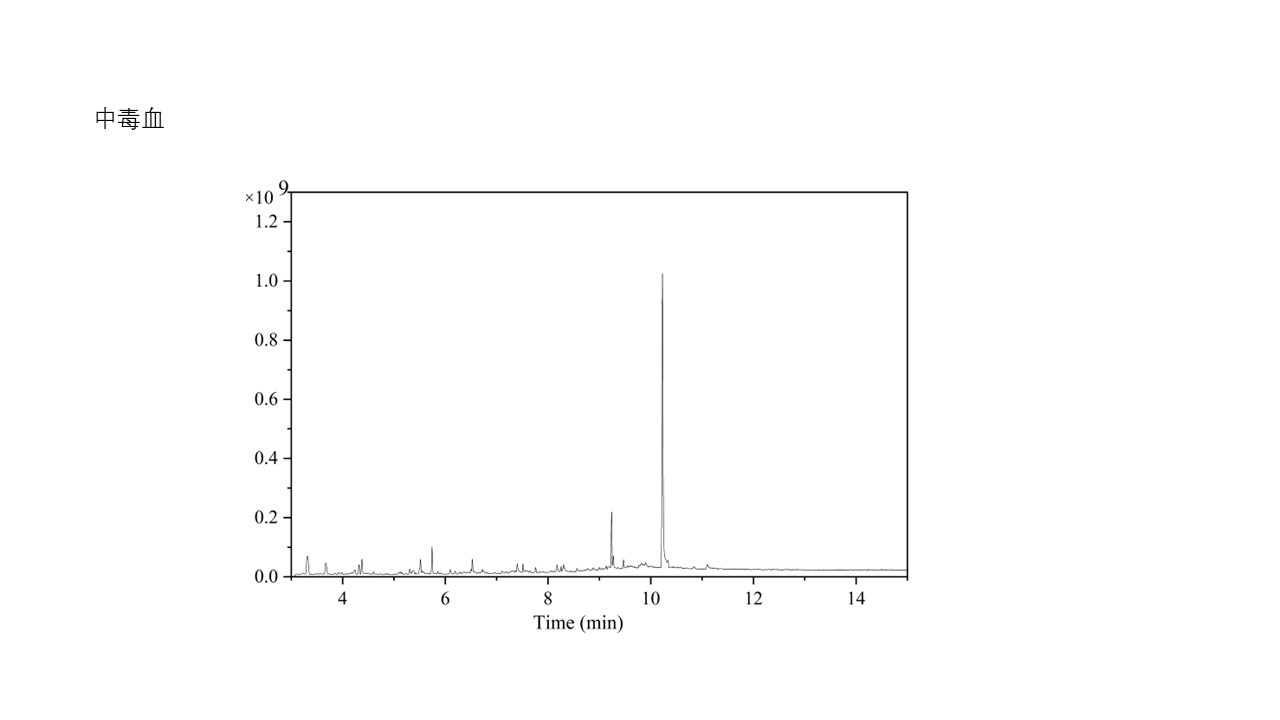


**Fig. S3** GC-MS total ion current chromatogram of blood in difenidol poisoned rats added SKF_525A_ (10.0 μg/mL). Difenidol *R*_t_: 10.23min; internal standard SKF_525A_ *R*_t_: 9.24min

**Fig. S4** Mass spectrogram and chemical structure of difenidol


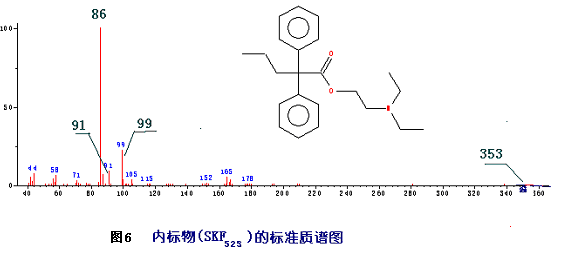


**Fig. S5** Mass spectrogram and chemical structure of SKF_525A_
